# Research on the Influence of the Material Removal Profile of a Spherical Polishing Tool on the Mid-Spatial Frequency Errors of Optical Surfaces

**DOI:** 10.3390/mi15050654

**Published:** 2024-05-15

**Authors:** Zhaohao He, Kuo Hai, Kailong Li, Jiahao Yu, Lingwei Wu, Lin Zhang, Xing Su, Lisheng Cai, Wen Huang, Wei Hang

**Affiliations:** 1 College of Mechanical Engineering, Zhejiang University of Technology, Hangzhou 310023, China; he_zhaohao@163.com (Z.H.); wlw15990434168@163.com (L.W.); 2 Sichuan Precision and Ultra-Precision Machining Engineering Technology Center, Chengdu 610200, China; 13568045419@163.com (L.Z.); suxinghit@126.com (X.S.); clsenrise011@163.com (L.C.); huangwenupc@126.com (W.H.); 3 Key Laboratory of Advanced Ceramics and Machining Technology, Ministry of Education, Tianjin University, Tianjin 300072, China; 4 School of Mechanical and Electrical Engineering, University of Electronic Science and Technology of China, Chengdu 611731, China; yjh_3166@163.com

**Keywords:** sub-aperture polishing, mid-spatial frequency error, convolution effect, material removal profile

## Abstract

Elastic spherical polishing tools effectively conform to the polishing surface and exhibit high efficiency in the removal of materials, so they are extensively used in the sub-aperture polishing stages of optical components. However, their processing is often accompanied by significant mid-spatial frequency (MSF) errors, which critically degrade the performance of optical systems. To suppress the MSF errors generated during polishing with spherical tools, this study investigates the influence factor of MSF errors during the polishing process through an analysis of the convolution effect in material removal. A material removal profile model is established, and a uniform removal simulation is conducted to assess the influence of different shape material removal profiles on MSF errors. Simulation and experimental results show that a Gaussian-like shape material removal profile is more effective in suppressing the MSF errors during polishing compared to the “W” and trapezoidal shape material removal profiles. In addition, based on the characteristics of the RMS decreasing in a serrated trend with the decrease in path spacing, a path spacing optimization method considering the polishing efficiency is proposed to improve the polishing efficiency while controlling the MSF errors, and the effectiveness of the path spacing optimization method is verified by comparing the MSF error at the maximum theoretical path spacing and the path spacing that is less than this. Finally, the path spacing optimization method is used to polish single-crystal silicon to further illustrate its practicality.

## 1. Introduction

Optical components are widely applied in fields such as precision instrumentation and optical imaging, including extreme ultraviolet lithography systems, space telescopes, high-power laser equipment [1,2,3,4,5,6], etc. With the rapid development of these areas, higher precision requirements for optical components have become necessary. Polishing is the final processing step for optical components and plays a vital role in achieving the ultimate precision of the component. With the advances in computer technology, computer-controlled optical surfacing (CCOS) technology has been successfully applied to the ultra-precision polishing stage of optical component manufacture [7]. Compared to traditional polishing methods, CCOS technologies ensure accurate material removal, better predictability of the results, and higher processing efficiency. Today, based on CCOS technology, various polishing methods, such as magnetorheological polishing [8], bonnet polishing [9], ion beam polishing [10], and abrasive jet polishing [11], have widely been employed in the manufacture of optical components, significantly improving the processing quality and efficiency. In the CCOS technology, the size of the polishing tool is much smaller than that of the workpiece, which ensures the convergence efficiency of the surface form to a certain extent, but introduces additional MSF errors during the polishing process [12,13]. The elastic spherical polishing tools are widely used in the sub-aperture polishing stage of optical components because of their effective fit to the polished surface and efficient material removal ability. However, more severe MSF errors are produced during the polishing process. MSF errors degrade the performance of optical components, such as by producing small-angle scattering and reducing image contrast [14], which greatly impacts the imaging quality of the optical system. Therefore, reducing the MSF errors produced during the polishing process is of great significance.

In the sub-aperture polishing process of optical components, it is commonly believed that the generation of MSF errors is due to the regular removal of materials along the polishing path by the polishing tool. Compared with the more regular path, the pseudo-random path direction is more variable, which allows for a certain degree of suppression of MSF errors on the polishing surface. Consequently, many scholars have researched pseudo-random paths. Dunn et al. [15] first proposed a universal pseudo-random path that has been widely applied in magnetorheological finishing and abrasive jet polishing, effectively suppressing MSF errors. Subsequently, some researchers applied various paths, such as maze paths, Peano paths, six-direction pseudo-random paths, and circular pseudo-random paths [16,17,18,19], in optical sub-aperture polishing, achieving different degrees of MSF error suppression. Despite the effective suppression of ripple errors through the use of pseudo-random paths, the variability in the direction of the paths can lead to significant velocity changes near the path turning points, which requires a high dynamic performance by the machine tools and is detrimental to the convergence of the polished surface’s form. Wan et al. [20] suggested that the mechanism by which pseudo-random paths suppress MSF errors involves the transfer of MSF errors to lower-spatial-frequency errors, which sacrifices the efficiency of surface form convergence to some extent. Frequent changes in the path direction can damage the machine tool. Hence, raster paths remain the most commonly used polishing paths. Another method to suppress MSF errors involves the use of small polishing pads for smoothing ripples. To smooth surface ripples effectively, small polishing pads require a certain level of stiffness; however, to ensure adequate conformity of the small pad with the workpiece, especially during the smoothing of curved components, the pad also needs to possess a certain degree of flexibility. Therefore, designing small polishing pads that meet the specific stiffness and flexibility requirements presents a challenge. Kim et al. [21] designed a rigid–conformal small polishing pad and proposed a parametric smoothing model to quantitatively describe the smoothing effects of the polishing tools. The flexible layer of this pad was made of a viscoelastic material, which can be considered a non-Newtonian fluid. During the polishing process, its unique rheological properties enabled better contact with aspheric surfaces and maintained sufficient stiffness under high-speed impact conditions, achieving a certain smoothing effect. Building on Kim’s work, Su et al. [22] introduced a parametric smoothing model to describe the smoothing of complex surfaces. Song et al. [23] proposed a time-varying smoothing model that included specific factors related to smoothing efficiency based on the parametric smoothing model and Preston’s equation. The smoothing efficiency of the rigid conformal polishing pad can be maximized by utilizing the time-varying smoothing model. Yu et al. [24] designed a rigid aspheric smoothing tool for aspheric and freeform surface smoothing and demonstrated its effectiveness in removing MSF errors on aspheric surfaces through comparative experiments with spherical smoothing tools. Hou et al. [25] proposed a predictable smoothing evolution model combining process parameters and error reduction factors and experimentally proved that the predictive model was consistent with the actual smoothing effect. However, smoothing is a time-consuming process and there is a high probability that the surface form of the workpiece will be damaged during the ripple smoothing process.

Although ample research has been conducted on the suppression of MSF errors during the sub-aperture polishing of optical components, the primary focus has been on optimizing the polishing path or improving the smoothing ripple performance of small pads to mitigate MSF errors, and little attention has been paid to the influence of the material removal characteristics of the polishing tool itself on the MSF error. Furthermore, some scholars have investigated the effect of different process parameters on the profile of the tool influence function (TIF) and analyzed the convergence efficiency of the TIF with different cross-section profile shapes to surface forms, but did not discuss its effect on the MSF error [26,27,28]. Therefore, this paper analyzes the formation mechanism of MSF error based on the convolution effect of material removal. A material removal profile model is established, and different shapes of removal profiles are used to simulate the MSF error distribution on the processed surface. In addition, a path spacing optimization method is proposed to improve the polishing efficiency while effectively controlling the MSF error.

## 2. Theoretical Analysis

### 2.1. Analysis of MSF Error Source

In the sub-aperture polishing of optical components, the amount of material removed is equal to the convolution of the tool influence function (TIF) with the dwell time along a specified polishing path, which can be expressed as follows [29]:

(1) H(x,y)=R(x,y)∗T(x,y)

where H(x,y) is the amount of material that is removed; R(x,y) is the TIF; T(x,y) is the dwell time at the dwell points along the polishing path; and ∗ is the convolution operation.

**Figure 1:**
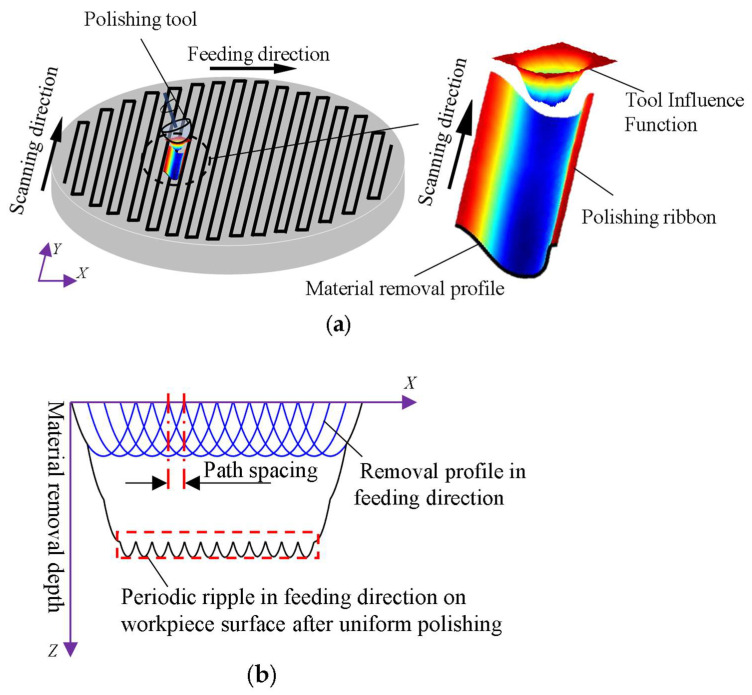
Analysis of MSF source. (**a**) Schematic diagram of polishing using a raster path; (**b**) periodic ripples in the feed direction.

The polishing tool moves continuously in the scanning direction and discretely in the feed direction during the polishing process, as shown in Figure 1a. The final material removal can be viewed as two parts. First, the TIF moves continuously along the scanning direction on one polishing path to form a material removal ribbon, in which no ripples are considered to be generated. Then, the material removal ribbons formed on different polishing paths are convolved and superimposed to form the final material removal. As shown in Figure 1b, due to the convolutional nature of material removal, the overlap of material removal ribbons on adjacent polishing paths leads to non-uniformity in the material removal. This generates a periodic ripple error, also known as an MSF error, in the scanning direction. In this paper, the material removal profile is defined as the cross-section of the material removal ribbon perpendicular to the tangential direction of the path, and it is obvious that the shape of the material removal profile directly affects the formation of the ripples.

### 2.2. Material Removal Profile Model

As mentioned above, the formation of ripples is closely related to the superposition state of the material removal profile. Thus, analyzing the formation of the material removal profile is of significant importance for the suppression of ripples.

According to the Preston equation [30], the material removal at the contact area between the polishing tool and the workpiece can be expressed as follows:

(2) dh(x,y)=KP(x,y)V(x,y)dt

where dt denotes the dwell time; P(x,y) denotes the pressure in the contact region; V(x,y) denotes the relative velocity between the polishing tool and the workpiece in the contact region; dh(x,y) denotes the depth of material removal during the dwell time dt; and *K* is the Preston coefficient.

**Figure 2:**
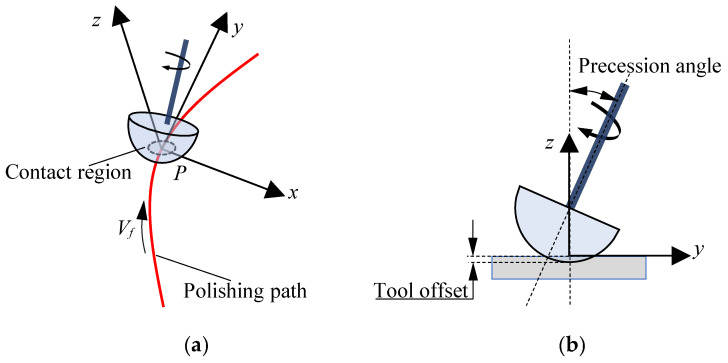
Positional relationship between the polishing tool and the workpiece. (**a**) Three-view schematic diagram; (**b**) axial schematic diagram.

**Figure 3:**
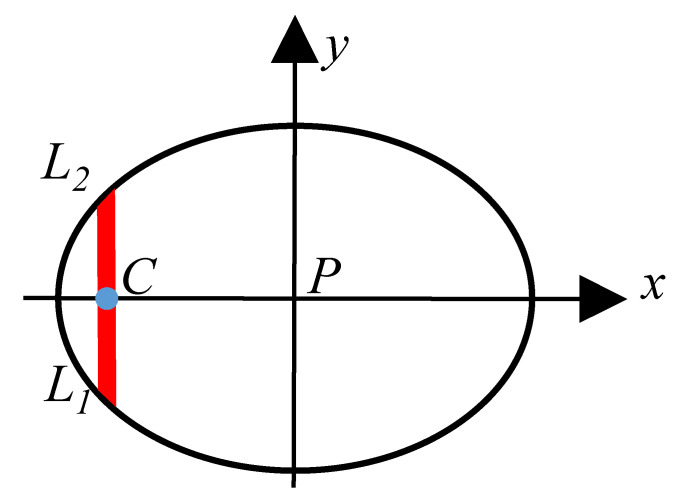
Local polishing area between polishing tool and workpiece.

The elastic spherical polishing tool moves along the polishing path with a given scanning rate $V_{s}$, the direction of the *y*-axis is the tangent direction of the polishing path, and the direction of the *x*-axis is perpendicular to the tangent direction of the polishing path, as shown in Figure 2. Figure 3 shows the schematic diagram of the local contact area between the polishing tool and the workpiece. When the polishing tool moves along the *y*-axis with a given scanning rate $V_{s}$, the distance traveled by the polishing tool during the period dt is:

(3) $$dy=V_{s}dt$$

Substituting Equation (3) into Equation (2) yields:

(4) $$dh(x,y)=\frac{KP(x,y)V(x,y)}{V_{s}}dy$$

Set point *C* for any point on the *x*-axis in the contact area, *L*_1_ and *L*_2_ are the end and start points of the polishing tool along the straight polishing path through point *C*, respectively. Then, the depth of material removal produced at point *C* is:

(5) $$h(x)=\int_{L_{2}}^{L_{1}}\frac{KP(x,y)V(x,y)}{V_{s}}dy$$

It is evident that Equation (5) is the material removal profile model.

Given a polishing path spacing of *s*, the 2D material removal profile *S* on the surface of the workpiece in the feed direction is expressed with the following equation:

(6) $$S(x)=\sum_{i=1}^{n}h_{i}(x-(i-1)s)$$

where $h_{i}$ denotes the removal profile on the *i*th polishing path.

In Equation (6), it can be seen that the overlap of the removal profile directly affects the generation of ripples in the feed direction. Therefore, the effect of the material removal profile and path spacing on the MSF error is simulated and analyzed in the next section.

## 3. Simulation Analysis

### 3.1. Simulation Analysis of the Influence of Material Removal Profile on MSF Errors

**Figure 4:**
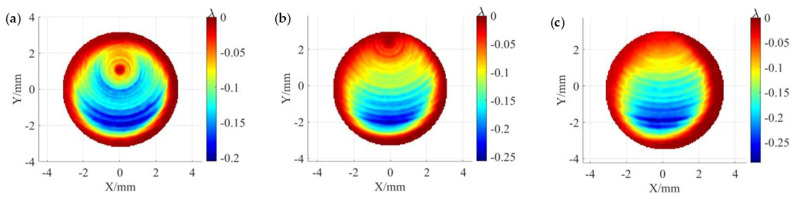
TIFs for different precession angles. (**a**) At 3°; (**b**) 7°; (**c**) 11°.

**Figure 5:**
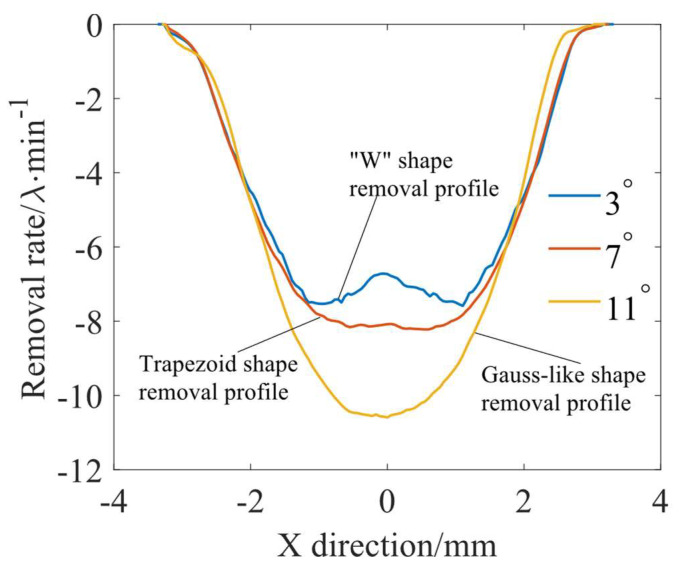
Material removal profile at different precession angles.

To obtain the TIFs required for the simulation, fixed-point polishing experiments were carried out under different processing parameters. The workpieces and polishing tools used in the experiments were 50 × 50 mm square planar-fused silica pieces and elastic spherical polishing tools with a radius of 20 mm, respectively. The polishing tool was bonded with a polyurethane polishing pad of 1 mm thickness, and the polishing fluid was a 3.5% CeO_2_ slurry with mass fraction. The specific processing parameters are shown in Table 1. The experimentally obtained TIF is shown in Figure 4, where *λ* is a unit wavelength of 632.8 nm. According to Equation (5), the TIF is calculated along the *Y*-axis to obtain the different material removal profiles, as shown in Figure 5, and the scanning rate adopted in the calculation is 30 mm/min. It can be seen that due to the difference in the precession angle, three different shapes of removal profiles appear, which are as follows: the “W” shape removal profile, the trapezoidal shape removal profile, and the Gaussian-like shape removal profile.

Based on the analysis presented in the previous section, it is clear that the formation of MSF errors is closely related to the removal profile and the overlapping condition of these profiles. Therefore, three different removal functions in Figure 4 are used to simulate uniform polishing under raster paths with different path spacings to analyze the effect of three typical material removal profiles on the MSF error. To ensure that the material removal rate of different TIFs in the simulation is consistent, and to avoid the effect of different material removal depths on the MSF error, the scanning rates at the 3°, 7°, and 11° precession angles are 6.2 mm/min, 6.4 mm/min, and 7.5 mm/min, respectively.

**Figure 6:**
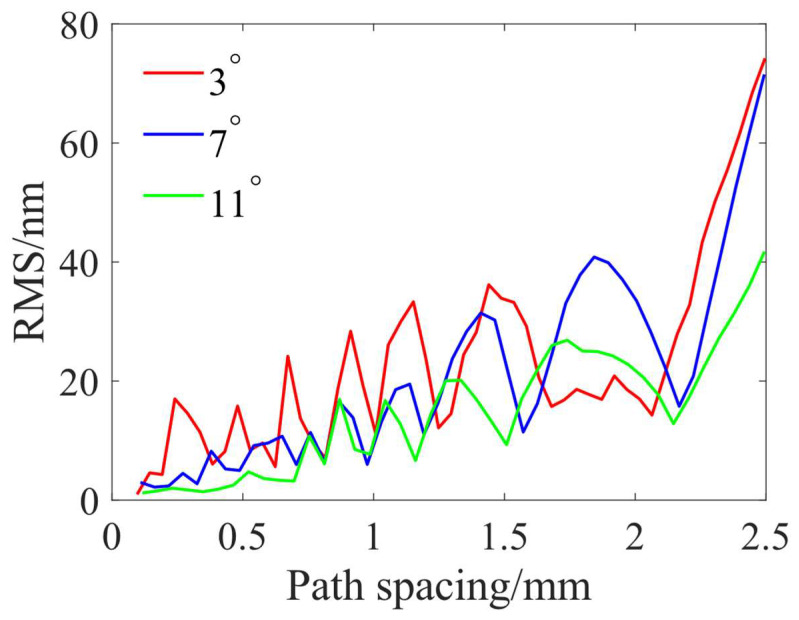
RMS obtained by simulation at different path spacings using TIFs at different precession angles.

According to Equation (1), the simulation is carried out for an area of 30 × 30 mm; to avoid the influence of the edge effect, the circular center area with a radius of 5 mm is extracted for the analysis. Figure 6 shows the root mean square (RMS) value obtained from the simulation of different removal profiles with different path spacings. It can be seen that as the path spacing decreases, the RMS with different removal profiles decreases as a whole. However, from the localized view of the curves in Figure 6, the RMS values do not decrease monotonically with the decrease in the path spacing but show a serrated change, which is due to the influence of the superposition complexity of the removed profiles on the adjacent polishing paths under different path spacings. Moreover, the RMS of the “W” shape removal profile at a precession angle of 3° is significantly higher than that of the trapezoidal shape removal profile at a precession angle of 7° and the Gaussian-like shape removal profile at a precession angle of 11°, and the RMS of the Gaussian-like shape removal profile, which is the smallest overall. This indicates that at the same path spacing, the Gaussian-like shape removal profile has the best material removal uniformity and can minimize the generation of ripples.

**Figure 7:**
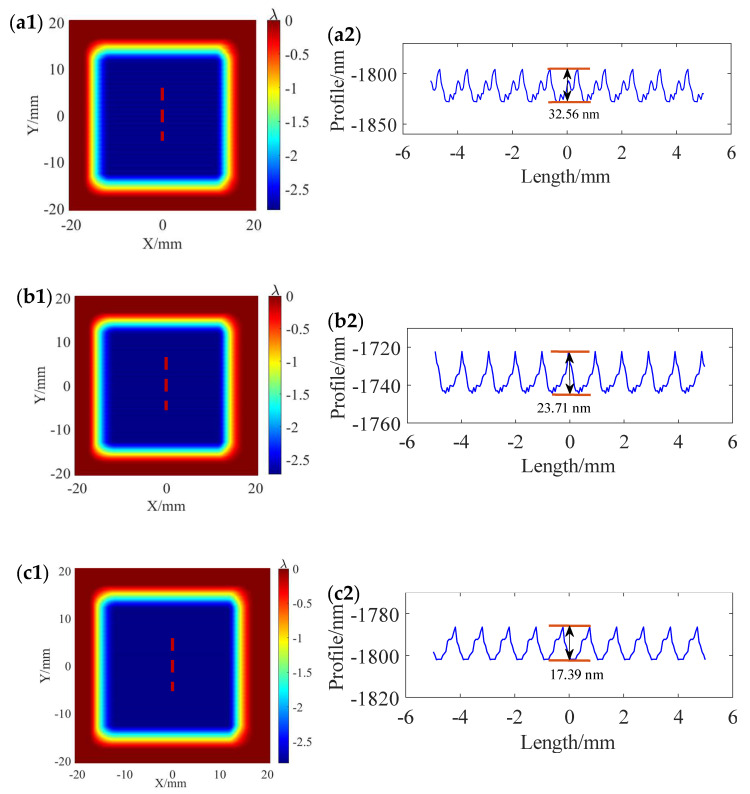
Surface morphologies and corresponding cross-section profile simulated at 1 mm path spacing using TIFs at different precession angles: (**a1**,**a2**) at 3°; (**b1**,**b2**) 7°; (**c1**,**c2**) 11°.

**Figure 8:**
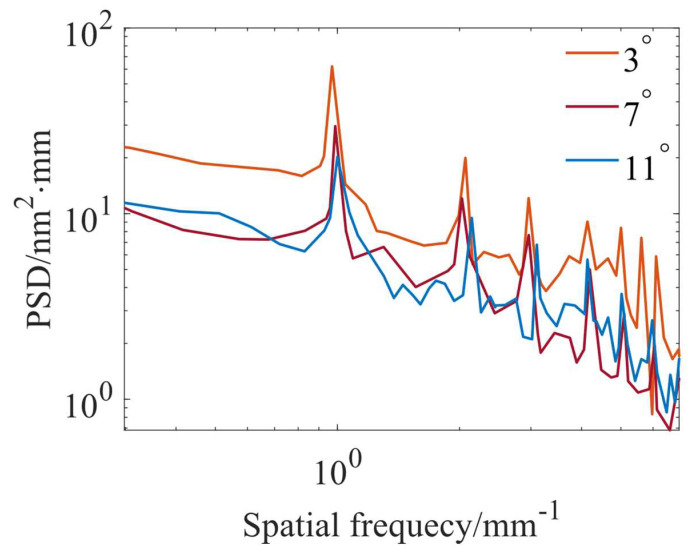
PSD curves obtained by simulation at 1 mm path spacing using TIFs at different precession angles.

To further illustrate the effect of different removal profiles on the MSF error, the power spectral density (PSD) curves obtained after a uniform polishing simulation with different removal profiles at a 1 mm path spacing are used for an example analysis. Figure 7 shows the material removal distribution calculated after uniform removal simulations with different removal profiles at 1 mm path spacings. As shown in Figure 7a2,b2,c2, the ripple heights obtained after uniform polishing using the “W” shape, trapezoidal shape, and Gaussian-like shape removal profiles are 32.56 nm, 23.71 nm, and 17.39 nm, respectively. The use of the “W” shape removal profile is effective in reducing the ripple height. Figure 8 shows the corresponding PSD analysis curves. From the PSD curves, it can be observed that the main peak appears at the spatial frequency position of about 1 mm^−1^, which corresponds to the 1 mm path spacing. In Figure 8, it can also be seen that the amplitude of the main peak under the “W” shape removal profile is the largest, followed by the trapezoidal shape removal profile, and the amplitude of the main peak under the Gaussian-like shape removal profile is the smallest. The simulation demonstrates that the use of the Gaussian-like shape removal profile can reduce the generation of ripples in the polishing process.

### 3.2. Path Spacing Optimization Method

**Figure 9:**
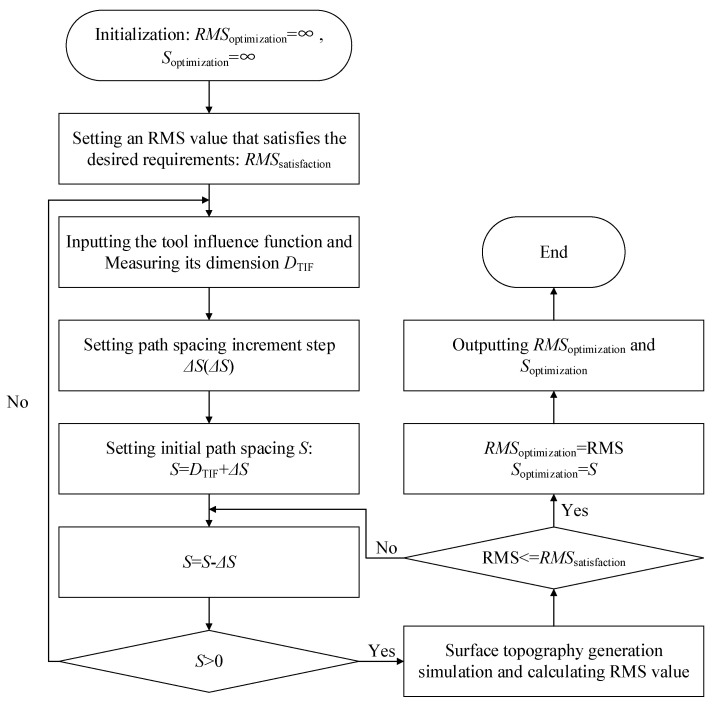
Path spacing optimization process.

From the above analysis, it can be seen that the generation of MSF errors can be suppressed using a smaller path spacing and a Gaussian-like removal profile through the control of the polishing precession angle. However, a smaller path spacing will increase the polishing time, and requires a higher dynamic performance and positioning accuracy of the machine tool, meaning that the path spacing cannot be set too small. In addition, as the path spacing decreases, the RMS shows an overall decreasing trend; however, it shows a serrated change locally. Even with a larger path spacing, a relatively smaller RMS can be obtained at some specific spacings, which provides the necessary conditions for optimizing the path spacing. Therefore, an optimal path spacing selection method considering polishing efficiency is proposed. Figure 9 shows the process of finding the optimal path spacing. To evaluate the MSF error, some scholars have introduced different metrics, such as RMS value [31], PSD [32], and peak-to-valley (PV) value [33]. In this process, the RMS is used to evaluate the MSF error, and the maximum path spacing that satisfies the desired RMS is found through the iterative decrease in the path spacing step by step so that the polishing efficiency can be improved under the premise that the MSF error is effectively controlled.

## 4. Experiments

### 4.1. Experiment Setup

**Figure 10:**
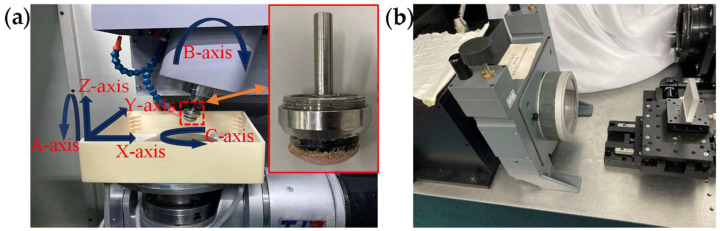
Experimental equipment. (**a**) A six-axis CNC machine with spherical polishing tools; (**b**) Zygo laser interferometer for form measurement.

To verify the effect of the removal profile on the MSF error and the proposed optimal path spacing selection method, eight 50 × 50 mm square planar-fused silica glasses with a relatively uniform initial surface form were used for uniform polishing experiments to analyze and compare the MSF error under different path spacings and different removal profiles. The experimental parameters are similar to the simulation parameters, and the specific experimental parameters are shown in Table 2. The polishing fluid used was a slurry with a 3.5% mass fraction of CeO_2_. The experiments were carried out on a six-axis CNC machine with a spherical polishing tool and a Zygo laser interferometer was used to measure the surface form of the workpiece, as shown in Figure 10. To facilitate the observation and analysis of the MSF error on the surface of the polished workpiece, a band-pass filter with a frequency range of 0.2–10 mm^−1^ was used to filter the surface form. To avoid the influence of edge effects, the center area of the workpiece with a range of about 10 × 10 mm was extracted for the analysis.

### 4.2. Influence of Material Removal Profile on MSF Error

**Figure 11:**
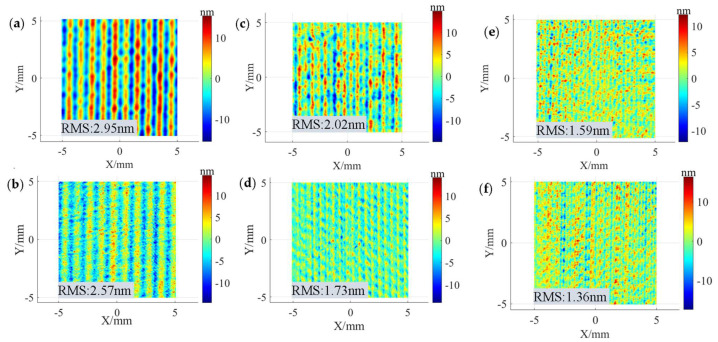
Surface morphologies obtained under different path spacings and precession angles: (**a**) 3°, 1 mm; (**b**) 11°, 1 mm; (**c**) 3°, 0.5 mm; (**d**) 11°, 0.5 mm; (**e**) 3°, 0.3 mm; (**f**) 11°, 0.3 mm.

**Figure 12:**
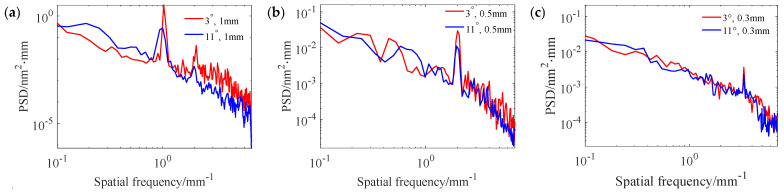
PSD curves under different path spacings and precession angles: (**a**) 1 mm; (**b**) 0.5 mm; (**c**) 0.3 mm.

In optical component sub-aperture polishing, the path spacing used is generally no larger than 1/4~1/3 of the size of the TIF. Therefore, the 0.3 mm, the 0.5 mm, and the 1 mm path spacings were selected for uniform polishing experiments to analyze and compare the influences of the “W” shape removal profiles and the Gaussian-like shape removal profiles on the MSF errors. The relevant experimental parameters are shown in the No.1 and No. 2 experiments in Table 2. The experimental results are shown in Figure 11. Figure 11a,b show the surface morphology after polishing with the “W” shape and Gaussian-like shape removal profiles at a 1 mm spacing, with an RMS of 2.95 nm and 2.57 nm, respectively. Figure 11c,d show the surface morphology after polishing with the “W” shape and Gaussian-like shape removal profiles at a 0.5 mm path spacing, with an RMS of 2.02 nm and 1.73 nm, respectively. Figure 11e,f show the surface morphology after polishing with the “W” shape and Gaussian-like shape removal profiles at a 0.3 mm path spacing, with an RMS of 1.59 nm and 1.36 nm, respectively. The experimental results show that the Gaussian-like shape removal profile is more conducive to RMS convergence than the “W” shape removal profile. Figure 12 shows the PSD curves orthogonal to the scanning direction of the polishing path under the corresponding conditions in Figure 11. From the figure, it can be seen that, compared with the “W” shape removal profile, under the same path spacing, the PSD curve of the Gaussian-like shape removal profile has a smaller peak amplitude at the characteristic frequency corresponding to the pathing spacing, and the PSD curve amplitude changes steadily at other frequency components, except the characteristic frequency. This indicates that the Gaussian-like shape removal profile can effectively reduce the generation of surface ripples compared with the “W” removal profile, which is consistent with the trend of the simulation results.

### 4.3. Validation of Path Spacing Optimization Method

The previous experiments and simulations have shown that the Gaussian-like shape removal profile can reduce the MSF error generated by the polishing process compared with the “W” shape removal profile and the trapezoidal shape removal profile. To further suppress the MSF error and ensure polishing efficiency, the polishing path spacing needs to be optimized. In the task of MSF error control within a reasonable range at the same time, a larger path spacing is required, if possible, to improve polishing efficiency. Here, it is assumed that the RMS in the MSF error range needs to be controlled below 2 nm. According to the simulation results in Figure 6 and the path spacing optimization method in Figure 9, the theoretical maximum path spacing for uniform polishing simulation using Gaussian-like shape removal profiles is about 0.4 mm under the condition that the RMS of the workpiece surface after uniform polishing simulation is less than 2 nm. In addition, the theoretical RMS of a uniform polishing simulation with a Gaussian-like shape removal profile at 0.2 mm path spacing is 1.67 nm. To verify the effectiveness of the path spacing optimization method, the surface MSF errors after uniform polishing using Gaussian-like shape removal profiles at these two path spacings are compared. The relevant experimental parameters are shown in the No. 3 experiment presented in Table 2.

**Figure 13:**
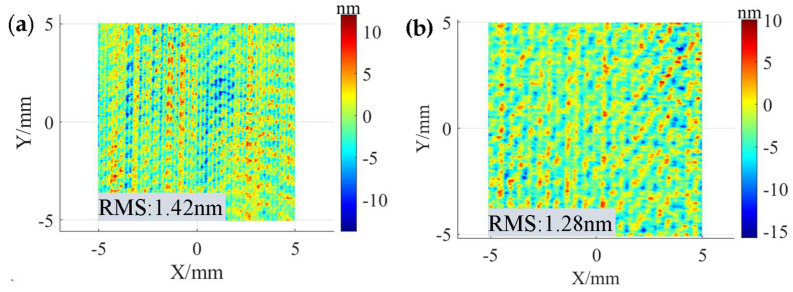
Surface morphologies obtained under different path spacings: (**a**) 0.2 mm; (**b**) 0.4 mm.

**Figure 14:**
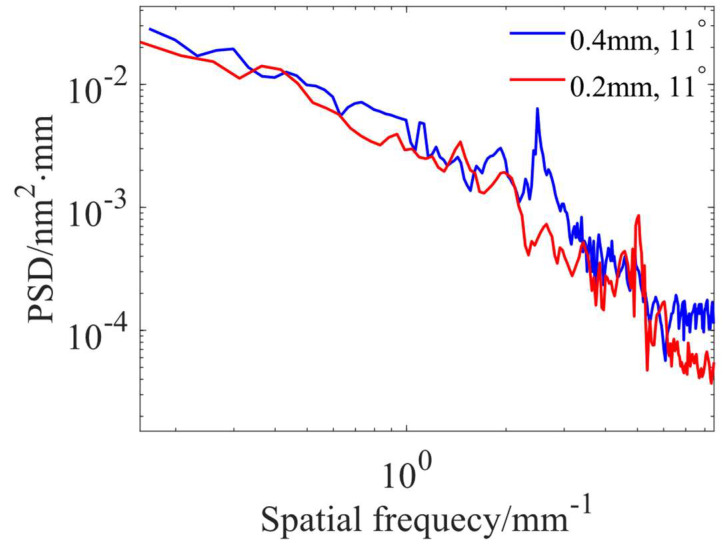
PSD curves under different path spacings.

The experimentally obtained surface morphology is shown in Figure 13. The corresponding PSD curves orthogonal to the scanning direction are shown in Figure 14. The RMS of the MSF range at the 0.2 mm path spacing is 1.42 nm, and the peak amplitude of its PSD curve at the 5 mm^−1^ characteristic frequency is about 8 × 10^−4^ nm^2^·mm. The RMS of the MSF range at the 0.4 mm path spacing is 1.28 nm, and the peak amplitude of its PSD curve at the 2.5 mm^−1^ characteristic frequency is about 6 × 10^−3^ nm^2^·mm. The RMS after polishing with both path spacings meets the required requirements, but the 0.4 mm path spacing should be preferred to ensure polishing efficiency. The effectiveness of the path spacing optimization method is experimentally verified.

### 4.4. Application of Path Spacing Optimization Method

**Figure 15:**
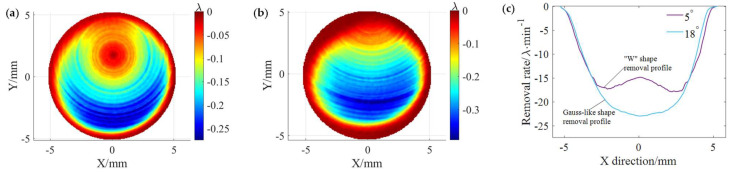
TIFs under different precession angles and corresponding material removal profiles: (**a**) At 5°; (**b**) 18°; (**c**) material removal profile at different precession angles.

In Section 4.3, the effectiveness of the path spacing optimization method was experimentally verified. In this section, polishing experiments are performed on single-crystal silicon to further illustrate the practicality of the path spacing optimization method. Before to the polishing experiments, TIF generation experiments were conducted on single-crystal silicon to obtain TIFs with two different shape removal profiles, as shown in Figure 15. The polishing solution used in the experiment was 3.5% CeO_2_ slurry with mass fraction, and citric acid was added to make its PH 5. The specific parameters of the tool influence function generation experiment are shown in Table 3. Here, it is assumed that the RMS within the MSF error range needs to be maintained below 1.5 nm. The path spacing was optimized according to the path spacing optimization method in Section 3.2, and the theoretical maximum path spacing for the uniform polishing simulation using the TIF with the Gaussian-like shape removal profile in Figure 15b was about 0.36 mm. Uniform polishing experiments were performed at a 0.36 mm path spacing using the TIFs of two different shape removal profiles in Figure 15. To ensure consistent material removal, the scanning rates for polishing using the TIF of the “W” shape removal profiles and the TIF of Gaussian-like shape removal profiles were 15.9 mm/min and 17.3 mm/min, respectively.

**Figure 16:**
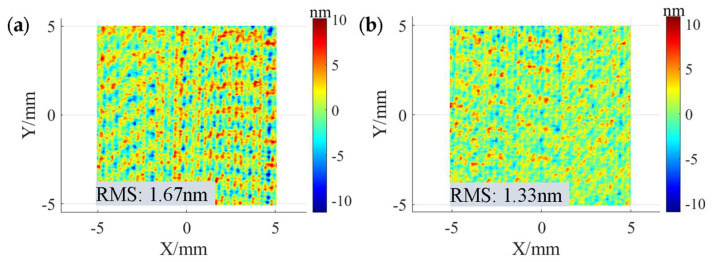
Surface morphologies after polishing under different precession angles: (**a**) 5°; (**b**) 18°.

**Figure 17:**
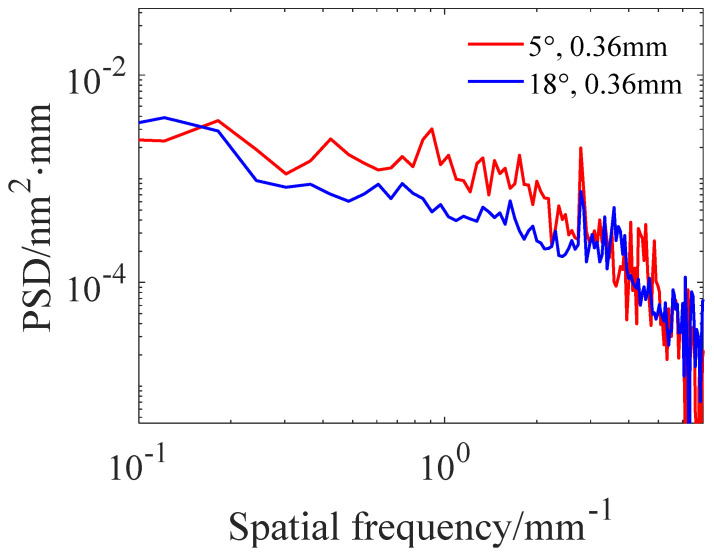
PSD curves under different precession angles.

Figure 16 shows the surface morphology after polishing using two TIFs with different shape removal profiles. The RMS after polishing using the TIFs of the “W” shape removal profile and Gaussian-like shape removal profile is 1.67 nm and 1.33 nm, respectively, which indicates that polishing at the maximum theoretical path using the Gaussian-like shape removal profile can keep the RMS in the MSF error range within the desired range. On the other hand, it can be observed from the PSD curves in Figure 17 that, compared with the “W” shape removal profile, the PSD curve of the Gaussian-like removal profile has a smaller peak at 2.8 mm^−1^ frequency, which also indicates that the use of the path spacing optimization method is effective in suppressing the MSF error on the polished surface.

## 5. Conclusions

Elastic spherical polishing tools have a high material removal efficiency and a good fit to the workpiece surface, and are especially suitable for polishing curved optical components. However, the polishing tool produces more severe MSF errors in the polishing process. This paper analyzes the source of MSF errors when polishing with elastic spherical polishing tools based on the material removal convolution effect, and suppresses the generation of MSF errors through the optimization of the material removal profile and the path spacing. The main conclusions of this paper are as follows:

(1) The formation mechanism of MSF error is analyzed based on the material removal convolution effect, and it is found that the material removal profile and path spacing have an important influence on the generation of MSF errors.

(2) A uniform polishing simulation was carried out using different removal profiles with different path spacings, and the results show that, compared with the “W” shape removal profile and trapezoidal shape removal profile, the Gaussian-like shape removal profile is more effective in minimizing MSF errors.

(3) Focusing on the characteristics that the RMS decreases in a serrated trend with the decrease in path spacing in the simulation analysis, a path spacing optimization method was proposed. The effectiveness of the path spacing optimization method is verified by polishing fused silica glass and single-crystal silicon, and it was shown that the MSF error can be effectively controlled even under a larger path spacing.

This paper provides process guidance for the suppression of MSF errors generated during the polishing process of spherical polishing tools.

## Figures and Tables

**Table 1:** Process parameters for generating TIFs.

Parameter	Value
Tool radius (mm)	20
Tool offset (mm)	0.2
Precession angle (˚)	3, 7, 11
Rotation rate (rpm)	300
Dwell time (s)	10

**Table 2:** Experimental parameters.

Experiment	No. 1	No. 2	No. 3
Tool radius (mm)	20	20	20
Tool offset (mm)	0.2	0.2	0.2
Precession angle (°)	3	11	11
Rotation rate (rpm)	300	300	300
Scanning velocity (mm/min)	6.2	7.5	7.5
Path spacing (mm)	0.3, 0.5, 1	0.3, 0.5, 1	0.2, 0.4

**Table 3:** Process parameters for generating TIFs.

Parameter	Value
Tool radius (mm)	20
Tool offset (mm)	0.6
Precession angle (°)	5, 18
Rotation rate (rpm)	300
Dwell time (s)	10

## Data Availability

The original contributions presented in the study are included in the article, further inquiries can be directed to the corresponding authors.
